# Toxicity of Glutathione-Binding Metals: A Review of Targets and Mechanisms

**DOI:** 10.3390/toxics3010020

**Published:** 2015-01-26

**Authors:** Federico Maria Rubino

**Affiliations:** LaTMA Laboratory for Analytical Toxicology and Metabonomics, Department of Health Sciences, Università degli Studi di Milano at “Ospedale San Paolo” v. A. di Rudinì 8, I-20142 Milano, Italy; E-Mail: Federico.Rubino@unimi.it; Tel./Fax: +39-02-50323034

**Keywords:** arsenic, cadmium, EdAG, enzymes, glutathione, lead, mass spectrometry, mechanism, metallothionein, mercury, nanoparticles

## Abstract

Mercury, cadmium, arsenic and lead are among priority metals for toxicological studies due to the frequent human exposure and to the significant burden of disease following acute and chronic intoxication. Among their common characteristics is chemical affinity to proteins and non-protein thiols and their ability to generate cellular oxidative stress by the best-known Fenton mechanism. Their health effects are however diverse: kidney and liver damage, cancer at specific sites, irreversible neurological damages with metal-specific features. Mechanisms for the induction of oxidative stress by interaction with the cell thiolome will be presented, based on literature evidence and of experimental findings.

## 1. Introduction

Several metals are toxic to humans exposed through occupational sources, environmental contamination, water and food. Upon chronic exposure, most of these metals accumulate in the body throughout human life and their biological effect contributes to shorten the natural lifespan of organs such as the kidney and ultimately determine their early failure [1]. However, the mechanisms through which individual metals target specific organs and cause a distinctive pattern of pathological signs is understood in limited detail.

The mechanism by which thiol-binding metals exert a toxic activity on cells and organisms is in general terms described as the consequence of “interaction with and inhibition of essential thiol groups of enzymes and proteins”, although the systemic toxicity of the metals is very different [2]. In particular, mercury is much more toxic with respect to other thiol-binding metals: as an indication, the DL_50_ values of some water-soluble salts are: HgCl_2_ (oral, rat): 3.7 micromoles/kg; CdCl_2_ (oral, rat): 473 micromoles/kg; Pb(OAc)_2_ (oral, dog): 914 micromoles/kg; ZnCl_2_ (oral, rat): 2381 micromoles/kg (MSDS Data Sheets accessed at: http://msds.chem.ox.ac.uk/).

Moreover, the observed effects on health and behavior are often specific to overexposure to specific metals. As an example, mercury, lead, cadmium and manganese all damage the central nervous system but with consequences of very different nature. Manganese causes a neurologic impairment resembling parkinsonism [3] or epilepsy [4]; cadmium causes a loss of olfaction due to selective cytotoxicity of olfactory neurons [5]; mercury and lead generate well-defined and different neurobehavioral syndromes. The disease generated by mercury, “*mercurial erethism*”, is a depressive occupational disease with specific symptoms and manifestations, of which a literary description is that in Lewis Carrol’s Mad Hatter, frequent still down to the 1950s, and caused in workers involved in hair-felting with mercury salt solutions [6,7]. The disease produced by exposure to tetraethyl-lead suddenly appeared in the 1920s, when the compound was introduced as the anti-knock component of “Looney gasoline”, and is characterized by hallucinatory and excitatory symptoms. According to recordings, an Italian worker heavily intoxicated in a tetraethyl-lead manufacturing plant expired standing on his deathbed and singing an opera *aria* of “La Traviata” [8].

Different chemical agents can trigger common mechanisms of toxicity at the level of organism, organ, tissue and cell. In the case of organic compounds, biotransformation steps are often necessary and their nature, mechanism(s), responsible enzymes and related polymorphisms are well characterized and the respective role in determining individual sensitivity to the effects of exposure at different levels is a well-established field of investigation.

In contrast, the selectivity and strength of metal ion binding to specific motifs and the subsequent reactivity derived from this interaction are not taken frequently into account in the effort to explain the differences in the toxic manifestations of most metals. Those for which the differences are more evident are in particular those showing an affinity for the sulphur atoms present in the biological structures, most often as the thiol(ate) functional group of cysteine, and the “soluble thiolome”.

**Scheme 1:**
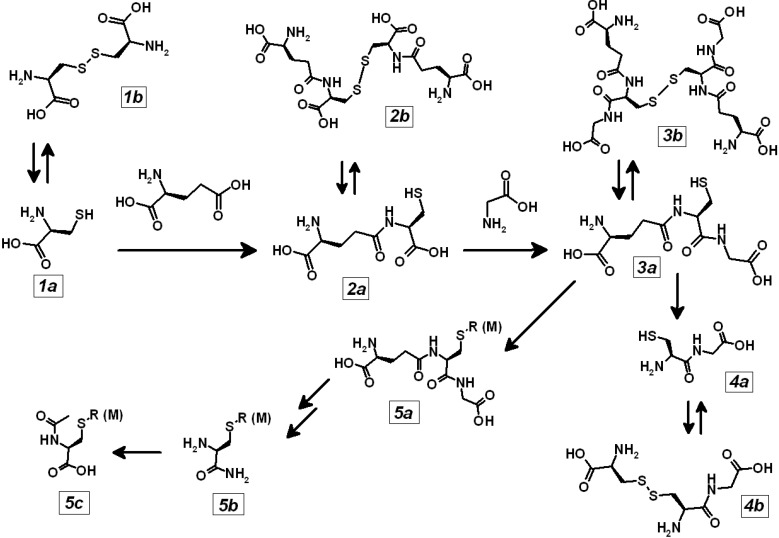
Simplified metabolic grid of the soluble thiolome.

The “soluble thiolome” is illustrated in Scheme 1 and operationally defined as the metabolic grid centered on the flux of the sulphur-containing amino acid cysteine (***1a***), on the production of the anabolic intermediate γ-glutamyl-cysteine (***2a***), of the abundant (pseudo-)peptide glutathione (***3a***) and of its catabolic product cysteinyl-glycine (***4a***). Each thiol can be reversibly converted from the “reduced” (“***a***”) thiol form of the cysteine sulphur atom into the “oxidized” (“***b***”) disulfide form.

Glutathione (***3a***) also reacts with organic electrophiles (**R**), such as several reactive metabolites of environmental contaminants and pharmaceutical drugs, and with metal ions of thiophilic elements (**M**) to yield the corresponding glutathione thioethers (***5a***), alkyl-cysteines (***5b***) and mercapturic acids (***5c***).

Glutathione biosynthesis occurs in several, but not in all eukaryotic cell types through a well-coordinated and feedback-modulated process that needs one essential amino acid, cysteine, and two others, glutamic acid and glycine, that most cells can synthesize themselves from the intermediates of core metabolism. Some cell types, among which the energy-ravenous and stress-sensitive brain neurons, cannot synthesize glutathione endogenously and rely on import from neighboring cells of the glia. Cells synthesize glutathione in the cytosol, rather than on ribosomes, and binding of the amino group of cysteine to the γ- rather than to the α-carboxyl group of glutamic acid makes the bond inert towards peptidases. The presence of a thiol group in glutathione is at the core of its biochemical behavior. One function of glutathione is to scavenge even-electron endogenous and exogenous electrophilic species, through the formation of thioethers (conjugation reaction). The corresponding reaction that occurs with some inorganic ions (thiol-binding metals and semi-metals, such as cadmium, mercury, lead and arsenic) is the core topic of this review. Another key role of glutathione is as an anti-oxidant and free radical scavenger role, particularly important in the mitochondria, where the catabolic conversion of nutrient substrates into carbon dioxide and water, with the concomitant recovery of chemical energy as ATP and of reductive power as NAD(P)H takes place. Free radical scavenging by glutathione takes place through the (formal) oxidation of its sulphur atom to the corresponding sulphinic acid (GS-OH). This intermediate form can react with the thiol group of another molecule of glutathione (to yield glutathione disulphide) or of a protein (to generate a glutathionylated protein).

The multiple functions and relations of the “soluble thiolome” with the functional and signaling redox proteome in several conditions of exposure to endogenous and exogenous conditions have been repeatedly reviewed as soon as new aspects are progressively unveiled and clarified [9].

Oxidative stress is the condition whereby the living organism is unable to cope with an excessive production of free radicals and reactive electrophilic species generated within the cellular metabolism as a consequence of exposure to several chemicals, among which some metals [10,11]. Although oxidative stress may not be the only cause of cell derangement and of disease, an ever-increasing number of disease conditions at several organs and systems, such as cancer, cardiovascular, neurodegenerative and endocrine diseases, are linked to this altered state.

**Scheme 2:**
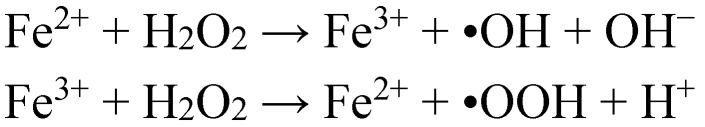
The general Fenton reaction as exemplified for iron ions.

Transition metals with two contiguous oxidation states, typically Fe and Cu, but also Co and Ni, are able to catalytically convert endogenous, naturally produced hydrogen peroxide into the hydroxyl radical through radical-chain mechanisms such as that long known as the Fenton reaction [12] (Scheme 2).

The hydroxyl and hydroperoxyl radicals are the eponyms of a family of Reactive Oxygen Species (ROS), which disruptively react with biological structures, such as unsaturated lipids of cellular membranes, the backbone and bases of DNA and essential chemical groups of catalytic and signaling proteins. Also high-valent chromium(VI) is able to generate reactive ROS during its conversion to the stable Cr^3+^ oxidation state [13] and this mechanism has been associated to the recognized carcinogenic activity of chromate salts [14].

Downstream to chemical damage, unrepaired biological structures can trigger cascading cellular events which may ultimately determine cell malfunctioning and death or unrestricted replication leading to cancer [15]. However, most Fenton-active metals do not preferably bind the thiolate group of glutathione and of proteins, but rather other nitrogen- and oxygen-containing functional groups, such as the imidazole nitrogen of histidine and the deprotonated alkoxy function of serine and threonine.

Other metals which are present in the environment as industrial pollutants are not able to generate free radicals by shuttling between contiguous oxidation states but it is known that organisms exposed either in their natural environment or in toxicological experiments show signs of oxidative stress, and some metals, such as As, Cd and Hg, are also carcinogenic to humans. Among these metals, the environmental pollutants Cd, Hg, Pb; biologically essential metals such as Zn and semi-metals such as As and Sb specifically bind the thiol group of glutathione to yield the corresponding mercaptide conjugates of [(GS^−^)_n_(Met^n+^)] stoichiometry [16,17,18]. These compounds have long been demonstrated to be the main excretion form of Cd [19] and Hg [20,21] in mammals and their toxicity is modulated by the nature of the metal-binding biothiol [22,23,24].

One critical point in understanding at the molecular level the toxicity of thiol-binding mineral elements such as As, Cd, Hg and Pb is that in *ex vivo*, *in vitro* assays toxicity occurs at micromolar concentration. This figure should be compared to the millimolar (*i.e.*, more than one thousand fold higher) concentration of glutathione alone, not including that of the thiol groups of other non-protein and protein components. Thus, metal concentrations that are high enough to be toxic may still be too low to deplete cytosolic glutathione pools to a significant extent on a stoichiometric basis. Moreover, experiments in isolated organisms, such as in yeast, show that exposure to elements such as cadmium causes an increase, rather than a decrease of glutathione concentration. The activity of several involved enzymes also increase, including those that allow an influx of sulphur (as inorganic sulphate, that is converted into homocysteine as the entry step into biological organic species), which is the rate-limiting element for the biosynthesis of biological thiols [25,26].

On the contrary, chronic exposure of humans to thiol-binding elements, such as arsenic, causes a decrease in the circulating concentration of glutathione and in a shift of the balance of disulphide, “oxidized”, to thiol, “reduced”, glutathione towards more oxidized status [27]. Indirect evidence of the efficacy of exposure to thiol-binding metals at environmental and occupational levels to induce oxidative stress and to decrease the glutathione pool emerges also from the study of biomarkers such as antioxidant enzymes and soluble oxidized metabolites [28,29,30,31].

The effect exerted by “one atom” of the toxic metal must thus be able to yield a multiplicative effect, such as that corresponding to the inhibition or inactivation of key enzymes that contain thiol groups as part of functional or regulatory mechanism. Moreover, an increasing number of biological functions are now known to involve glutathione, such as that in the biosynthesis of iron-sulphur clusters of mitochondrial enzymes [32,33].

As will be discussed, one mechanism which may connect thiol-metal binding to oxidative stress-mediated toxicity is specific of Hg, the cysteinyl-glycine bound form of which is able to deplete glutathione reservoirs by a catalytic process [34].

Humans and animals exposed to Hg accumulate the metal in several organs under the chemical form of highly insoluble but possibly bio-reactive metal sulphide nano-particles. Although their formation mechanism is still obscure, such body inclusions are common also in lower organisms such as plants and micro-organisms exposed to Pb, Cd and Zn [35].

A hint to their possible formation is to consider that the thioethers of reactive electrophiles with glutathione, cysteine and *N-*acetyl-cysteine can undergo in the kidney a β-elimination reaction to yield a thiol compound and a reactive, electrophilic dehydro-alanine derivative. The *N-*acetyl-cysteine thioethers, also known as mercapturic acids, are the excreted metabolites of industrial toxic organic chemicals [36] and this mechanism is considered to play a role in the kidney carcinogenesis by halogenated alkenes. A similar biochemical mechanism occurring on metal thiolates would generate also metal sulphides, as demonstrated by mass spectrometric studies.

In this article, we review the mechanisms through which some metals and semi-metals of environmental, public health and occupational concern, and endowed with a chemical affinity to the thiolate form of sulphur present in glutathione and in cysteine-containing proteins may exert their toxic effect(s) in the living cell. The aim of this selective compilation of a large body of research is to highlight the chemically plausible mechanisms through which some metals that are unable to react by the “radical” pathways related to the Fenton or Haber-Weiss reaction, are nevertheless able to generate a physiological damage by triggering oxidative stress through their reactivity towards the soluble thiolome pool.

Information deriving from such disparate fields as earth and environmental sciences, biomedicine, biochemistry and chemical biology, physical chemistry is employed to the aim of building as comprehensive as possible framework to understand the toxic properties of the metals based on the unifying mechanism(s) of their interaction with biologically crucial thiolate ligands.

## 2. Metal Conjugates of Thiol Amino Acids and Peptides

**Scheme 3:**
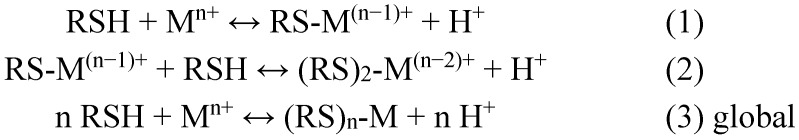
Formation of metal-thiolate conjugates.

Molecular compounds of stoichiometry [RS_n_(M^n+^)] are formed by reaction of metal ions and RSH thiolate ligands according to the equilibrium reactions of Scheme 3. The thiol-binding metals are not only those best-known for their thiol-binding ability, such as Mercury(II), Cadmium(II), Lead(II), Zinc(II), Arsenic(III and V), but also many more, such as Copper(I), Chromium(VI to III), Silver(I), Gold(I and III), Ni(II and III), Co(I and III), Al(III). The ligands are the thiolate-containing amino acids such as cysteine [16], the di- and tri-(pseudo)peptides belonging to the biochemical pathway of glutathione, the oligomeric glutathione correlates in the plant kingdom known as phytochelatins and some thiols of pharmacological or other practical utility, such as *N-*acetyl-cysteine (NAC).

Sometimes a selenium atom, in the amino acid seleno-cysteine, replaces the sulphur atom of the cysteine thiol group, which is mostly present as the active site of the enzyme peroxiredoxin and only in a minor fraction as the free amino acid.

**Scheme 4:**
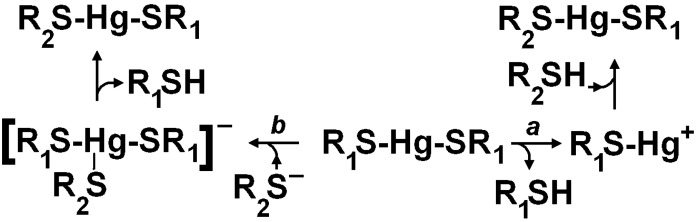
Mechanisms for thiol exchange of bis-thiolato-mercury(II) conjugates.

The process is dynamic, and involves also the thiolate groups of proteins such as serum albumin and haemoglobin and appears as the main mechanism for the distribution of thiol-binding metals from the absorption site throughout the body and towards the active sites where the toxic action is developed at the molecular level. The occurrence of these equilibria between the different thiol-bound forms of mercury, including the neurotoxic methyl-mercury, and of cadmium has been demonstrated to occur as exemplified in Scheme 4 for mercury, through Nuclear Magnetic Resonance experiments both of biomimetic models [37,38,39] and of intact erythrocytes [39,40,41,42].

The conjugates of glutathione with mercury and with methylmercury [43,44,45,46,47] have been identified in the bile [48] and in the urine of experimental animals dosed with the metals and their absorption in the kidney and processing by the cells of the proximal tubule have been investigated [21].

Binding of cadmium to the same classes of bio-molecules has been studied with a variety of analytical techniques, including mass spectrometry [16,18]. This phenomenon has been demonstrated *in vivo* [19,49], in erythrocytes [39,40] and in bio-mimetic models [37,50,51].

For several other biologically relevant metals, thiol-bound forms were postulated to form, or actually highlighted by *ex vivo in vitro* experiments that were performed mainly on whole blood or in isolated red blood cells.

In particular, Arsenic is a semi-metal with a complex ambivalent biochemical behavior: it is both a recognized human carcinogen and a curative anti-leukaemia drug and in both phenomena, its binding with cellular bio-thiols such as glutathione and with protein thiol groups is essential. It is known that As(III) species are much more toxic than those of As(V) and that biological methylation can enhance or reduce the toxicity of the different forms.

**Scheme 5:**
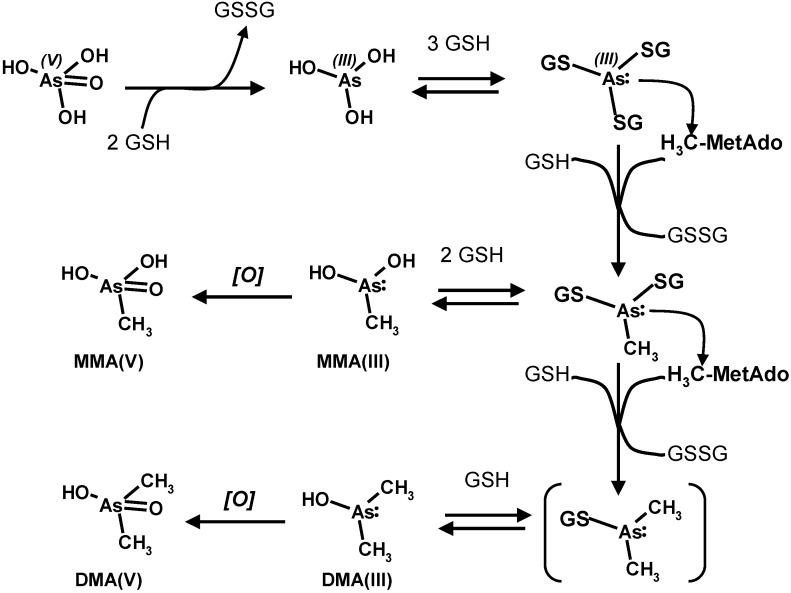
Glutathione-mediated biotransformation of arsenate. Adapted and integrated from Reference [57]. Copyright 2005, The Pharmaceutical Society of Japan.

As illustrated in Scheme 5, the complex cellular metabolism of arsenic is characterized by the reduction of As(V) to As(III) through an even-electron redox cycle which involves as the counterpart a bio-thiol such as glutathione and the formation of a *tris-*glutathionyl-As(III) species. The sequential methylation of As(III) by *S-*adenosyl-methionine to yield in turn the mono-, and di-methyl derivatives (and further the incorporation of the third methyl group and the generation of the haemolytic toxin trimethyl-arsine) also involves (“costs”) the oxidation of a further equivalent of “reduced” glutathione. The presence of glutathione in these intermediate conjugate forms of the different methylated forms of arsenic allows these molecules to be exported out of the cells by the ATP-binding cassette multidrug resistance proteins (MDR-C) system [52,53,54,55,56]. Moreover, the toxic and carcinogenic end-metabolite dimethylarsonic acid (MMA(V)) also reacts with glutathione to yield a conjugate with a high cytolethality [57].

It is likely that some steps of the biotransformation of arsenic that involve the formation of glutathione-bound species can occur also involving individual cysteine thiol groups or pairs of vicinal thiol groups in proteins, such as the catalytically essential thiol groups of enzymes and the regulatory elements of effector proteins that can be modulated by reversible glutathionylation (see further).

Platin-based anti-cancer drugs extensively bind to thiol groups in plasma, RBC and to intracellular peptides and proteins [58,59,60,61,62]. It is likely that this phenomenon plays a role both in detoxification (*i.e.*, in efflux from cancer cells as the cause for their chemo-resistance) and in the mechanisms responsible for the dose-limiting kidney toxicity of cisplatin and for its years-long terminal half life [63,64,65].

It is long known that cobalamin binds thiols through a strong and stable interaction of the formal cobalt(III) center with the thiolate anion of several biothiols such as glutathione [66]. Glutathionylcobalamin (GS-Cbl) is the predominant intracellular form of cobalamin [67] and possibly that most readily absorbed [68].

Another very complex picture of the interaction of metal centers with organic ligands in the pathway that leads to metal-caused biological impairment is that of Chromium, the high-oxidation state form of which, chromate or Cr(VI), is a known human carcinogen [14]. Chromate anions, that enter the cells through the sulphate anionic channel, meet the two intracellular reducing agents, glutathione [69] and ascorbate [70]. Cr(VI) species bind glutathione and other cysteine ligands *in vitro* and *in vivo* to yield several intermediate forms with different chemical stability, where the metal center has formally all the oxidation numbers between VI and III [71,72]. This phenomenon also occurs when cells take up particles of insoluble chromate pigments, such as Lead chromate [73]. However, notwithstanding the generation of free radicals, such as HO^.^, observed by EPR spectroscopy both *in vitro* and *in vivo*, the mutagenicity of the chemical reduction processes of Cr(VI) and thiols is too weak to explain the extent of damage to DNA [69]. The key step in the induction of mutagenic effects by Cr(VI) has been found to be the formation of Cr(III)-containing complexes between DNA and proteins [74,75].

The studies performed in subjects with chronic exposure to arsenic [27], or with clinically relevant intoxication by mercury [76], and in experimental animals intoxicated by mercury [77], cadmium and lead [78] show that the levels of some indicators of oxidative stress are displaced towards a pro-oxidative condition. The employed indicators include the GSH/GSSG ratio, reactive organic electrophiles (TBARS), enzymes such as catalase, glutathione peroxidase and SODs, protein carbonyls, urinary 8-hydroxy-2'-deoxyguanosine.

In considering the results of epidemiological studies, it should be considered that systemic compensation (homeostasis) could be acting to counter the biochemical effect of the toxic agent(s) [31]. Moreover, the several indicators of oxidative stress have different biochemical meaning and time course and at best, few of those available are measured in each study, and at a limited number of time points.

## 3. Oxidative Stress from Even-Electron Metal-Thiol Conjugates: A Case for Mercury

Oxidative stress is loosely defined as a condition of impaired physiological balance between the energy-producing oxidative metabolism of nutritional carbon substrates, oxygen supply to the cells and the concentrations of antioxidant compounds, which is reflected at the biochemical level into an imbalance of the dynamic physiological ratio of “oxidizing” and of “reducing” processes and related molecules [11]. At the chemical level the oxido-reductive (redox) status of a biological system can be quantitatively expressed through the relative levels of some biological indicators such as soluble thiols, nucleotide cofactors and other molecules which coexist in the organism as the pair of their “reduced” and “oxidized” form. In this case, the equilibrium redox potential of the “reduced” and “oxidized” form of the pair can be calculated through the Nernst equation and can be employed as a synoptic indicator of the status of the biological system [79]. This picture is entirely based on a thermodynamic interpretation of biological phenomena and has been recently challenged [80]. Flohe’s objection points out that the rate constants for the spontaneous reactions of inter-conversion of thiol disulfides are negligibly small, with respect to those necessary to explain biological processes, and therefore the intervention of the enzymatic systems is of a crucial importance in determining the behavior of a biological system.

Several review articles [11,81,82] discuss the causal relationship between an impairment of the homeostasis of redox-active metal ion and the irreversible transformation of biological constituents, such as proteins, lipids and the DNA strand, into oxidation products, and of the following biochemical impairment to the onset of a wide array of diseases.

Some forms of damage to biochemical structures occur from the reaction of chemically reactive (usually) small molecules with the nucleophilic centers of proteins and of DNA, that generate stable addition products, commonly referred to as “adducts” [83]. Some metals, especially those like mercury and arsenic, that generate strong bonds with the thiol group of cysteins, can impair the catalytic function of enzymes or modify the signaling of thiol-based molecular nano-switches [11,84].

In other cases, the reactive forms of oxygen (singlet oxygen, the superoxide and hydroxyl radicals, hydrogen peroxide), that are not coped with by the natural detoxifying mechanisms, can react with biological structures and impair their physiological function. The consequent derailment of the cellular behavior can thus lead towards apoptosis (a natural damage-limiting self-digestion of critically damaged cells, [85]), necrosis (an auto-sustaining, propagating tissue damage) and towards neoplastic transformation (an uncontrolled, invasive cellular growth).

Mercury is among the most toxic metals and an ubiquitous contaminant of the environment, due to its release from mining, from the combustion of fossil fuels, from industrial uses which are now being progressively restricted [86] and from other human activities such as artisanal exploitation of low-grade gold ores. Mercury is able to exert damage at several organs and systems where signs of an impaired energy metabolism and of oxidative stress can be highlighted, in particular as an imbalance of the physiological ratios of cysteine-containing peptides and proteins such as the soluble glutathione metabolome. However, well before the advent of the human kind, ancestral organisms were exposed to mercury released from volcanic eruptions. This has triggered the evolution of resistance mechanisms whereby the water-soluble toxic form of mercury, the Hg^2+^ cation (either aquated or coordinated to ligands such as chloride), is reduced back to the mono-atomic elemental form, the atoms of which are volatile and can be eliminated from the organism back into the environment [87]. The process is carried out by the mercuric reductase multi-enzyme complex. The genetic information for the several enzymes and transporters involved is packed into a long strand of bacterial DNA (a “*gene cassette*”), which in turn can be transmitted as a whole from one microbial cell to others through a genetic mechanism known as horizontal gene transfer, thus spreading antibiotic resistance throughout the pool of environmental micro-organisms. It is intriguing that mercury and antibiotic resistance genes can be localized in micro-organisms isolated from individual slices of a stratified core sample of sphagnum, a deposit of since-undisturbed decayed plant organic matter dating back 2000 years [88]. The key detoxication enzyme is MerA, the mercuric ion reductase, and also a second enzyme, MerB, is present in the gene cassette, and its activity is to split the carbon-Hg bond in organomercurials such as the potent, naturally occurring neurotoxic agent methylmercury chloride and the synthetic chemical disinfectant phenylmercuric acetate [89,90,91].

Briefly, Mercury(II) ions present in the environment cross the microorganism’s cellular membrane and are scavenged by MerA, a homo-dimeric enzyme which sandwiches the Hg^2+^ between a pair of catalytically essential cysteine thiol groups, to make a protein-embedded mercury(II)-bis-thiolate. The two-electron reducing agent is NADH and the enzyme requires a FAD cofactor close to the active-site Hg-binding cysteines, which operate through a mechanism possibly similar to that of the enzyme glutathione reductase (*vide infra*) [92,93,94]. The active site of the enzyme hosts two pairs of cysteines, one of which (^2^°^7^C^212^C and ^628^C^629^C, in *Bacillus* sp. RC607 MerA) binds mercury(II) ions and acts through a complex mechanism involving rapid thiol/thiol exchange to carry the bound ion to the site of reduction. This latter process occurs by reversible oxidation of a pair of cysteine residues, release of elemental Mercury(0) and subsequent reduction of the oxidized inactive enzyme by NADPH [95,96].

As far as known, only microorganisms have developed this defensive mechanism to get rid of toxic Mercury present in their environment. Eukaryotes lack this capacity and in particular complex animals can excrete absorbed Mercury only with difficulty, therefore most of the dose accumulates over time in long-half-life forms, from which the metal can be released and reach biochemically-impairing levels and thus exert toxic effects.

The glutathione-bound form of Mercury(II) is one of the most abundant forms of biochemically available Mercury in the body. The—at least partial [24,97]—structural and chemical similarity to physiological bio-molecules can explain the ability of thiol-bound Mercury(II) species to cross biological membranes by using the physiological transport systems of the natural substrates [21] and its ability to undergo chemical modifications operated by the enzymes which process bio-molecules such as those of the soluble thiolome. In fact, Wei and coworkers showed that there is at least indirect evidence that *bis-S*-glutathionyl-mercury(II): (a) is biochemically formed from exogenously administered mercury in exposed biological systems; (b) is a substrate of γ-glutamyl-transferase in full analogy with the behavior of other GSH adducts of xenobiotics [98].

To understand the chemical and biochemical behavior of molecules, reactivity studies can be performed *in vivo*, *ex vivo in vitro*, *in vitro* under biomimetic conditions and, recently, also through the study of the unimolecular decomposition of isolated molecules, as ions in a mass spectrometer. This latter technique allows us to investigate some molecular properties and to measure thermodynamic and kinetic parameters that may not be obtained as easily in other conditions. It may be especially noted that, in the active site of enzymes, the contribution of solvation from water is usually minimal and thus the information gained by exploiting some sophisticated techniques of modern mass spectrometry may better contribute to understanding biochemical mechanisms.

In particular, unimolecular decomposition of selected biomolecular precursor ions affords second- or higher-generation fragments in the mass spectrometer: this is the array of techniques referred to as “tandem mass spectrometry” or MS-MS [99]. This technique allows us to perform two tasks:

(a) to identify the chemical structure (“connectivity”) of the fragments, from which that of the precursor molecule can be inferred (structural analysis) [17,18,100], and
(b) to insight the kinetic and thermodynamic parameters which govern the formation of each fragment, as proxy of the phenomena that occur in solution and that play a role in the biochemical behavior of simple and complex biomolecules [34,101].

Among the properties of biomolecules which have been measured by these mass spectrometric techniques are the gas-phase basicity of amino acids [102,103,104,105] and of DNA nucleotides [103,106,107,108] and the reduction potential of the disulfide-thiol pairs of amino acids and peptides of the soluble thiolome [101].

One technique which has gained much interest among those of tandem mass spectrometry is that referred to as “*Energy-Resolved (tandem) Mass Spectrometry*” (ERMS). In this technique, collision-activated decomposition (CAD) is performed by impinging precursor ions accelerated at well-defined values of translational (kinetic) energy on a stationary target gas. At the core of the ERMS experiment is the collection of an array of collision-activated decomposition tandem mass spectra (CAD-MS-MS) of a molecular precursor ion, each obtained at a defined value of collision energy, to observe the fall of the intensity of the precursor and the gradual rise of the intensity of the generated fragments. From the obtained appearance curves the minimum value of collision energy at which a specific fragment can be generated are extrapolated and can be used as proxies of the actual thermodynamic parameter (ΔG_form_) which describes the ease of formation of a given species and its stability towards decomposition. This technique can be implemented in simpler instruments, such as triple-quadrupole mass spectrometers (those most commonly used for organic analysis in forensic toxicology, in diagnostic clinical chemistry and in metabolomic studies), or in state-of-the-art dedicated instruments, known as Guided-Ion Beam Mass Spectrometer(s) (GIBMS) [109].

The study by ERMS of some thiol-bound forms of metals has allowed underscoring some remarkable differences in their unimolecular decomposition, which can in turn be related to the physico-chemical properties of the bound metal and to their chemical and biochemical behavior in solution [110]. The gained information has allowed to explain some phenomena observed in toxicological studies performed at the level of cell cultures and of isolated tissue and organ preparations, and to finally propose an explanation for the peculiar toxicological behavior (*i.e.*, for the much higher toxicity) of Mercury among thiol-binding metals.

In particular, the comparative ERMS study of glutathione, glutathione disulfide and the glutathione-bound forms of the metals mercury, lead, cadmium and zinc highlighted several similarities of the unimolecular fragmentation to the known biochemical processes undergone by the molecules in solution.

First, all compounds undergo loss of the residue of γ-linked glutamic acid as pyroglutamic acid, although with different intensity, but with very similar onset energy between the physiological substrates of the enzyme, glutathione and glutathione disulfide and metal-bound glutathione conjugates. This result strengthens the biochemical evidence that several glutathione-bound metals are substrates of the enzyme γ-glutamyl-transpeptidase (GGT), the catabolic enzyme of the GSH-mercapturate pathway. In particular, the treatment with the enzyme of glutathione-metal conjugates isolated from the bile of experimentally intoxicated animals was used as an evidence of their possible connectivity in the earlier toxicological studies [43,47].

What shows the highest degree of difference between the different glutathione metal conjugates is the formation and further decomposition of the [GS-M]^+^ bound forms with a single glutathione unit. In particular, the mercury conjugate is the only one for which a decomposition pathway leaves the metal in the elemental form (neutral loss of Hg^0^) and the glutathione component in the “oxidized” sulfenium status.

**Figure 1:**
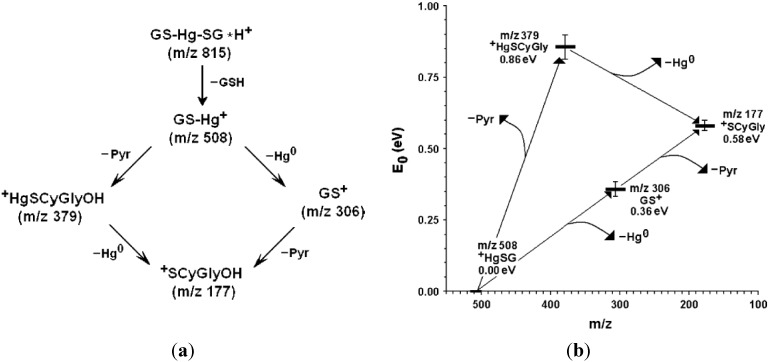
(**a**) Partial sequence of decomposition reactions of protonated *bis-*glutathionyl-mercury(II) (re-elaborated from Reference [34]); (**b**) Relative energy levels of the ion species reported in Figure 1a and starting from mono-glutathionyl-mercury(II) at *m*/*z* 508 (re-elaborated from Rubino 2006). Re-elaborated from Reference [34]. Copyright 2006, John Wiley & Sons, Inc.

Moreover, the energetic study of the observed decompositions shows that loss of elemental mercury from the glutathione-bound form is endoergonic (it needs some amount of collisional energy) while from the fragment derived from loss of the glutamic acid unit it is spontaneous. The sequence of decomposition reactions and the relative energy levels are reported in Figure 1.

This mass spectrometric behavior agrees with and justifies several independent lines of evidence which derive from chemical, biochemical and pharmacological studies.

First, the conjugate of mercury with cysteinyl-glycine is poorly stable in water and quickly decomposes, leaving a black residue of elemental mercury and almost total conversion of cysteinyl-glycine to its disulfide (our unreported observation).

Second, not only organic glutathione thioethers but also the metal conjugates can be enzymatically cleaved by the enzyme γ-glutamyl-transpeptidase (or γ-glutamyl-transferase, GGG). This has been demonstrated by the observation that the glutathione conjugate of the anti-cancer drug cisplatin, is enzymatically converted to the corresponding cysteinylglycine compound [59]. This enzyme was also long suspected to have a GSH oxidase activity, since a production of cysteinylglycine disulfide from GSH was observed [111]; this hypothesis was discarded by demonstrating that the measured disulfide was really an artefact due to rapid oxidation of the released thiol dipeptide [112]. The fact that cysteinyl-glycine is the most reducing thiolome compound [101] plays a fundamental role in the toxicology of mercury.

Third, a closer understanding of the cellular toxicity of mercury results from experiments performed on tubular kidney preparations to which synthetic Hg-biothiol conjugates were administered. The toxicity of exogenously administered Hg(SCyGly)_2_ towards renal proximal tubule cells was found to be higher than that of either (GS)_2_Hg or of (CyS)_2_Hg. When the degradation of GS_2_Hg to Hg(SCyGly)_2_ was inhibited by treatment with a specific inhibitor of GGT, this did not decrease Hg uptake, but decreased its cytotoxicity [98]. Also another experiment, performed in cell culture, showed that mercury is slightly more toxic in the presence of HSCyGly than of GSH [113].

Last, the treatment of mercury-intoxicated mice with *N-*acetyl-cysteine (NAC) enhances rather than reduces the renal toxicity of mercury [114]. This observation is at first paradoxical, considering that NAC is a well-known antidote for glutathione-depleting toxicants such as paracetamol overdose and that NAC yields mercury conjugates [17].

The reason for occurring this phenomenon (reduction of CySGly- but not of GS-bound Mercury(II)) in solution and in the biological environment of cells is reasonably a consequence of the low reducing electrochemical potential of CySGly (at −0.282 V the most reducing one of the whole soluble physiological thiolome). This value has to be compared to those of cysteine (−0.246 V), of glutathione (−0.205 V) and of NAC (−0.271 V, comparable to that of CySGly) [101]. There is a relationship of acidity and nucleophilic character of thiols with their disulfide-thiol oxidoreductive potential, by which those where the thiol group is more acidic, such as *N-*acetyl-cysteine are also those in which a more nucleophilic character of the thiolate sulphur also makes them more reactive in thiolate-disulfide exchange and therefore more effective as reducing agents [115,116]. In gas-phase isolated ions, it is the presence of the γ-glutamyl carboxyl group in the GSH conjugate that stabilizes the binding of mercury(II) to the thiolate ligand by a dipolar interaction, thereby preventing its loss as a neutral metal atom. This behavior is in accordance, although not necessarily in a causal relationship, with the higher reduction potential of glutathione.

**Figure 2:**
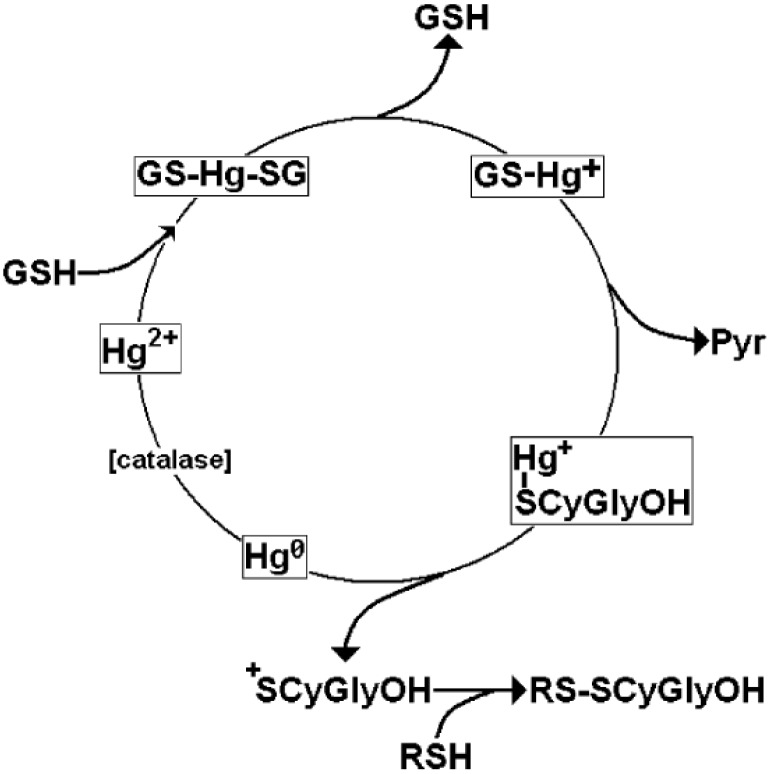
Proposed catalytic cycle for the depletion of the cellular pool of reduced glutathione by Mercury Re-elaborated from Reference [34]. Copyright 2006, John Wiley & Sons, Inc.

The scheme of Figure 2 describes a chemical-biological mechanism for the generation of intracellular oxidative stress that is caused by a continuous depletion of the cellular “reduced” glutathione pool by “catalytic” doses of Mercury.

This mechanism is proposed as the results of the reinterpretation of toxicological and biological experiments in the light of our physico-chemical measurements of the fundamental properties of the involved molecules, the conjugates of the thiol-binding metals with the soluble and protein thiolome, and of the corresponding disulphides.

Glutathione-bound Mercury(II) triggers the catalytic depletion of the cellular GSH pool by a catalytic mechanism, which involves the presence of Mercury as the glutathione conjugate. The first necessary step is the enzymatic removal of the glutamic acid residue from the glutathione-Mercury(II) conjugate to yield the Mercury(II)-cysteinyl-glycine conjugate. This product releases Mercury in its reduced form within the cellular environment, itself transforming into an oxidized sulfenium, sulfinic acid or disulfide form, which is highly reactive towards “reduced” thiol groups. In turn, this phenomenon can triggers a catalytic mechanism for the oxidation of the cellular thiol pool [117,118,119] and of oxidative stress when the homeostatic compensation is overwhelmed by an excessive depletion of the reduced thiol pool, as is the case for cells, such as neurons, that depend on neighboring glial cells for their supply of essential thiol precursors. This phenomenon can be caused by the fact that the released elemental mercury can be re-oxidized in the intracellular compartment by several enzymes, such as catalase [120,121] and re-enter the catalytic GSH-depleting cycle, ultimately leading to a displacement of the intracellular redox potential towards less negative, pro-apoptotic or necrotic values.

**Figure 3:**
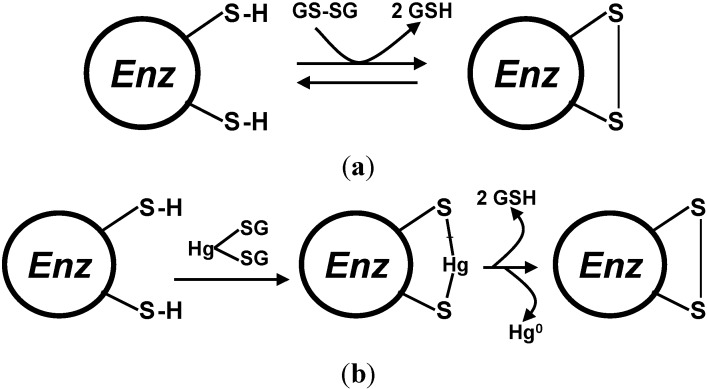
Possible mechanisms for the inhibition or inactivation of enzymes by glutathione-bound mercury. (**a**) is the physiological mechanism for enzyme action or regulation by the glutathione/glutathione disulfide redox pair; (**b**) is the interaction of glutathione-bound mercury with the vicinal thiol groups with intermediate transfer of mercury to the vicinal thiol pair, followed by oxidation of the thiols to an intramolecular disulfide and reduction of mercury(II) to the elemental form.

A possible mechanism that can lead to oxidation of vicinal thiol groups by reduction of glutathione-bound mercury(II) ions is described in the scheme of Figure 3.

It is conceivable that this general mechanism can explain Mercury-induced oxidative stress not only through a continuous depletion of the reduced glutathione pool, but also by interfering with enzymes operating through active sites based on vicinal thiol groups or which are regulated by glutathionylation of sensing cysteine residues. The first step of this mechanism can operate on those enzymes, which can thus be inhibited reversibly by also other thiol-binding metals different from mercury.

A different mechanism that can also lead to the irreversible inactivation of the enzymes is described and discussed in the following section.

## 4. Oxidative Stress from Even-Electron Metal-Thiol Conjugates: A Case for Arsenic, Cadmium and Lead

The mechanism proposed above for the generation of oxidative stress by mercury only holds due to the positive electrochemical reduction potential of mercury in water but cannot be generalized to other divalent cations of metals such as cadmium and lead, as well as for trivalent arsenic, which share with mercury a peculiar affinity to the thiolate sulphur functional group. Experimental toxicology studies in several animal species however show that these metals cause a decrease in the level of the measured thiols in the target tissues of the treated organisms [11] and generate oxidative stress, as measured by the several currently employed biomarkers. Since in particular the decrease of “reduced” thiols (most often glutathione) cannot be mechanistically caused by the same metal-centered mechanism that has been highlighted above for mercury, alternative biologically plausible pathways need to be searched for.

The study of the mass spectrometric fragmentation of “heavy” metal-biothiol conjugates has been instrumental in highlighting the key feature of thiol-bound mercury in its conjugates and is again the key to propose a more general mechanism for thiophilic glutathione-bound cations. Several more lines of evidence derive from *in vitro* bio-mimetic studies and from experimental toxicology studies of inorganic and organic chemicals. Their synoptic interpretation allows proposing a chemically plausible unified mechanism for the production of oxidative stress from electrochemically silent metal ions.

A crucial piece of evidence comes from the study of the deprotonated molecules of “heavy” metal-biothiol conjugates (*i.e.*, their prevailing ionic form under physiological conditions) by tandem mass spectrometry, which show a peculiar behavior, complementary to that observed for the positively charged corresponding species.

**Figure 4:**
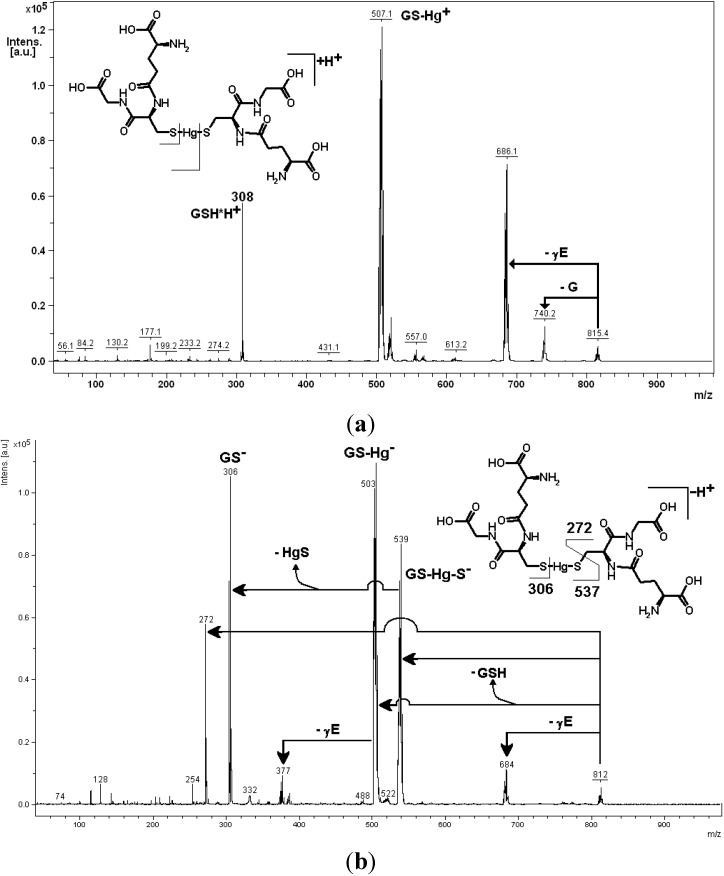
Unimolecular decomposition of protonated (**a**) and deprotonated (**b**) *bis-*gluthathionato-mercury(II) obtained in a MALDI-ToF-ToF instrument (sinapinic acid matrix; 15 kV collision energy).

**Scheme 6:**
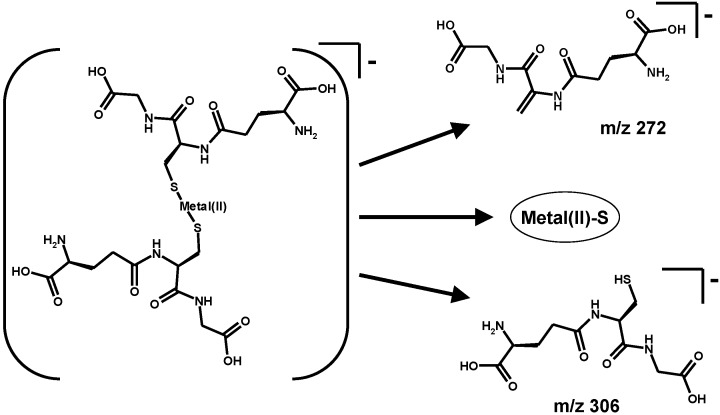
Decomposition of deprotonated metal(II) glutathione conjugates as observed by mass spectrometry.

A key example is shown in the spectrum reported in Figure 4b for the conjugate of mercury (original, unpublished results from our laboratory). The proposed decomposition pathway is shown in the inset of the spectra and in Scheme 6.

The gas-phase deprotonated molecule decomposes to yield the metal sulphide (as a neutral fragment; the arithmetic difference between two ion signals, in this case *m*/*z* 539 and 306) and an analogue of glutathione (fragment ion at *m*/*z* 272). This fragment ion contains the sulphur-deprived motif of dehydroalanine electrophilic in place of the cysteine residue (also referred to in the literature as EdAG; this acronym for “γ*-glutamyl-dehydroalanyl-glycine*” should not be confused with that, totally unrelated, for Erythroid Differentiation-Associated Gene, EDAG).

The occurrence of this pathway in metal-glutathione conjugates has been little studied by mass spectrometry, since positively charged molecules are most often studied. The observation of metal sulphides as neutral fragments in the unimolecular decomposition of bio-organic ions is relatively rare, and only one case has been very recently reported in a study of lead-binding metallothionein [122] (see below).

**Scheme 7:**
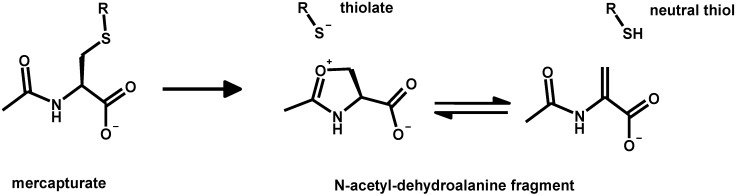
Mass spectrometric fragmentation of deprotonated mercapturates.

Deprotonated (negatively charged) molecules of structurally similar *N-*acetyl-cysteine (“*mercapturate*”) conjugates of organic electrophilic metabolites analyzed by mass spectrometry show the formation of ion fragments derived from the generation of a neutral fragment corresponding to acetamino-acrylate and of a thiolate anion. This mass spectrometric behavior, which is described in Scheme 7, is exploited as the best general strategy to analyze mercapturates by liquid chromatography and tandem mass spectrometry in metabolomic studies [123,124].

In principle, it is a more instrumentally sophisticated version of the long-employed test for urinary thiols excretion based on alkaline decomposition of urinary glutathione and mercapturic conjugates and on the measurement of the released thiols as a whole with the Ellmann reaction.

The employed decomposition pathway is analogous to that leading to the dehydro-alanine (acrylate) glutathione analogue (*m*/*z* 272 in Figure 4b). The mechanism formally parallels the β-elimination reaction catalyzed by PLP-dependant enzymes which accept cysteine thioethers as substrates and likely play a role in the renal toxicity and carcinogenicity of halogen-alkenes through their urinary metabolites [125].

**Scheme 8:**
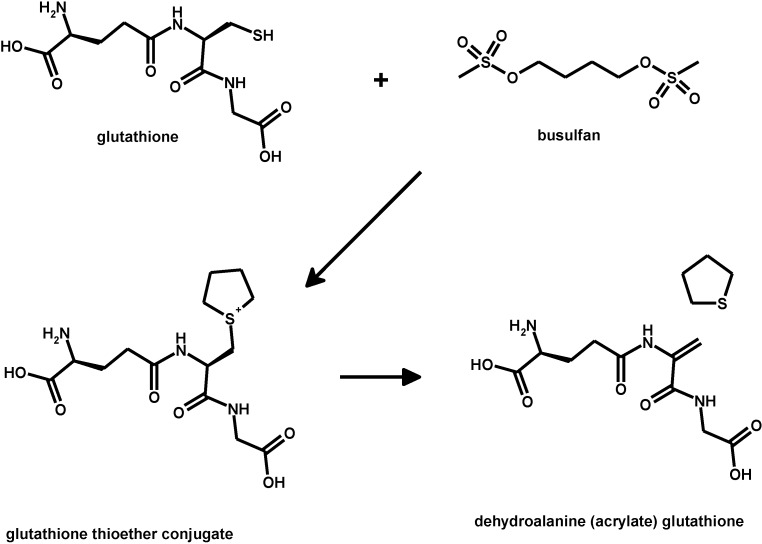
Bioreactivity of busulfan with glutathione.

To understand the possible role of this mechanism in the toxicity of metals and of organic compounds at the molecular level, it may be considered that the same endogenous electrophile can be generated *in vivo* from the bioconversion of the glutathione conjugate of some organic compounds. One well studied case is that of busulfan (as shown in Scheme 8) [126], an anti-cancer drug now widely employed to suppress lymphocytes and blood cell stem precursors prior to performing life-saving bone marrow transplantation in malignant diseases and in inborn blood diseases [127,128].

Once formed, the dehydro-alanine (acrylate) glutathione analog binds to glutathione*-S-*transferase GSTA1-1 [126]. Inactivation of the detoxifying enzyme glutathione*-S-*transferase has been observed in cells exposed *in vitro* to busulfan and, because of the inactivation of the enzyme, the level of free electrophilic species in the tissues of the exposed organisms can increase to levels that irreversibly damage the cell, which is deranged to apoptotic or necrotic death.

The chemical mechanism by which glutathione*-S-*transferase is inactivated by the glutathione acrylate analog is likely based on the presence in the tertiary structure of the enzyme of a docking site where glutathione is bound and its free thiol group is deprotonated by a facing amino acid which acts as a strong proton acceptor [129]. The thiolate cysteine sulphur then reacts with any reactive electrophile, which is able to dock in its own turn to the appropriate site of the enzyme. In most human GST isozymes the amino acid acting as the proton acceptor is a tyrosine, the phenol function of which is itself deprotonated by a neighboring strongly basic amino acid residue. For instance, as learned by X-ray crystallographic studies of complexes of the enzymes with glutathione alkyl thioethers as non-covalent inhibitors, in human α class glutathione transferase A1-1 the sulphur atom forms a hydrogen bond to the hydroxyl group of a tyrosine and to the guanido group of an arginine [130,131,132].

Two synthetic “des-thio” analogues of glutathione, one featuring a serine hydroxyl group in place of the cysteine thiol, the other being the alanine analogue, devoid of any polar or chemically reactive group in the place of cysteine were synthesized and their efficiency as competitive inhibitors of glutathione-*S*-transferase iso-enzymes 3-3 and 4-4 was tested. Both compounds could dock into the glutathione-recognizing domain of the enzymes, the serine analogue showing a higher affinity than the locally apolar alanine analogue [133].

Based on the displayed structures, and in particular with that of the glutathione conjugate of etacrynic acid [132], which is itself a Michael acceptor, it is well conceivable that glutathione acrylate can dock into the glutathione activating pocket of the GST enzymes and irreversibly bind to the essential tyrosine phenolate function, thus inactivating the enzyme.

**Scheme 9:**
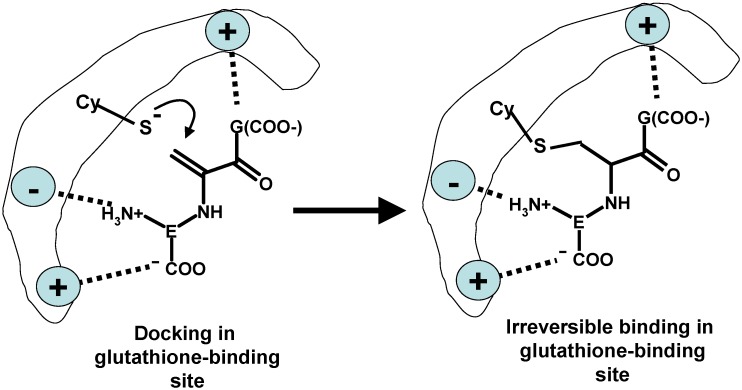
Outline of the mechanism for enzyme inactivation by dehydro-alanine (acrylate) glutathione.

Thus, dehydro-alanine (acrylate) glutathione can irreversibly bind to and inactivate other key enzymes for which glutathione is a substrate or a regulatory cofactor and thus possess a glutathione-docking motif in their tertiary structure. For this interaction to occur, the structural requirement in the enzyme is the presence of a nucleophilic amino acid side-chain close to the glutathione-docking motif and in a crucial position to interact with the electrophilic motif of glutathione acrylate, as shown in Scheme 9.

## 5. Oxidative Stress from Inactivation of Glutathione-Related Enzymes: A Non-Comprehensive Catalogue

Inhibition or irreversible inactivation of glutathione-regulated enzymes is a key step in the derangement of cellular equilibria, possibly leading to oxidative stress. Thiol (“reduced”) glutathione and disulfide (“oxidized”) glutathione often act as actuators of molecular nano-switches that activate, regulate or inhibit the activity of enzymes as a response to the presence of different levels of reactive species, such as ROS and electrophilic organic metabolites. Their action signals the cell to modulate its response, through the recruitment of endogenous coping mechanisms, among which are an increased synthesis of antioxidants and of electrophile quenchers, the transcription of the necessary gene products and the production of reducing cofactors such as NADH/NADPH and ascorbate. A specific mechanism by which glutathione thiol and glutathione disulfide operate to this aim is the reversible thiol(ate)-exchange mechanism, through which “an excess” of glutathione thiol regulates in an inhibitory sense the biological pathways which produce reducing cofactors or conditions by binding to sensing, conformation-determining disulfide bonds of proteins in a process termed reversible protein glutathionylation [134]. Conversely, “an excess” of glutathione disulfide is produced by more oxidizing conditions and regulates in a stimulatory sense the biological pathways, which produce reducing cofactors or conditions by binding free thiol groups of the sensing units of regulated proteins and setting free an equivalent of glutathione thiol.

### 5.1. Glutamate-Cysteine-Ligase

The biosynthesis of glutathione is tightly controlled at several levels, starting from DNA transcription to enzyme expression and activity modulation. The key enzymes in the biosynthesis of glutathione are the cytosolic **glutamate-cysteine-ligase** (GCL; EC 6.3.2.2), which catalyzes the formation of γ-glutamyl-cysteine, and glutathione synthase, which catalyzes the condensation of γ-glutamyl-cysteine and of glycine. Both enzymes consume ATP but only the former enzyme is subject to feedback inhibition by glutathione, which competes with glutamic acid for the active site.

It is known that, in rat and human GLCL holoenzyme, cystamine completely inactivates rat GLCL activity by interaction with a thiol group that is thought to be at or near the l-glutamate-binding site in the wild enzyme [135,136,137,138] and also in a recombinant mutant (hGLCLC-C553G) where one cysteine group far from the active site was selectively inactivated [139]. This finding points at the possibility that also thioether-forming electrophiles can inactivate the enzyme if they are able to dock to the enzyme, possibly if they possess a glutamic acid residue in their structure. Although direct evidence has not been obtained to time, it is likely that dehydro-alanine (acrylate) glutathione can inactivate the enzyme at its active site.

Moreover, physiological GCL (holo-enzyme) is composed of two subunits, respectively the 73 kDa GCLC and the 28 kDa GCLR subunit. The heavy GCLC is both the catalytic unit and the regulatory site of GSH feedback inhibition, while the light subunit regulates the activity of the holo-enzyme by modulating the affinity of the two regulatory sites of GCLC, that for glutamate and that for glutathione thereby making the enzyme more efficient and less sensitive to feedback inhibition. Site-directed mutagenesis experiments show that cysteine-553 in human GLCLC is involved in heterodimer formation between the catalytically active GLCLC and the auxiliary, activity-enhancing GLCLR unit, which likely occurs through a disulfide bond [139]. Formation of thioethers at cysteine-553, such as with the lipid peroxydation biomarker 4-HNE inhibits the formation of the GCLC(S-S)GCLR holo-enzyme and results in a decrease of GSH production. For the same reason above, it is possible that dehydro-alanine (acrylate) glutathione can inactivate the binding capacity of GCLC to form the holo-enzyme.

**Scheme 10:**
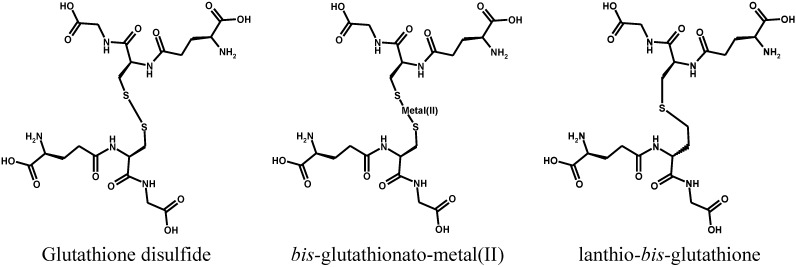
Structural similarity of glutathione disulfide, metal(II)-bis-glutathionate and lanthionine glutathione.

The consequence of both modifications is to lower glutathione synthesis and cellular levels, thus making this essential co-factor less available to exert its multiple biological functions. Glutathione is the essential co-factor or the product of several oxido-reductases, which link the NAD(P)H pool (that is produced by metabolic carbohydrate conversion to carbon dioxide), to the reduction of ROS generated by the electron flux of oxidative phosphorylation. The latter process immobilizes into ATP the chemical free energy generated by the successive steps of the mineralization of the carbon substrates of glycolysis and of the Krebs cycle occurring in the mitochondria. Although the glutathione pool of mitochondria is less than 20% of the total cell GSH, its role in the organelle is crucial to the maintenance of cell homeostasis and to routing disabled cells into apoptotic or necrotic death. The main involved enzyme families are: glutathione reductases, glutaredoxins, glutathione peroxidases, peroxiredoxins, and glyoxalases. A wealth of information on the structures, catalytic mechanisms and biological roles of these enzymes has been recently reviewed [129]. One main finding on the structural side is that the glutathione-hosting site is highly conserved, and uses the negatively charged carboxylate functions of the glycine and glutamic acid residues of glutathione as anchors for substrate recognition by positively charged amino acid residues, as well as negatively charged residues to hold the protonated amino group of the glutamic acid residue. The correct recognition position docks the disulfide group of GSSG close to the appropriate thiolate group of a deprotonated catalytically active cysteine residue, which is the actual reducing agent of the disulfide group, operating by trans-sulfuration. It is worth noting that glutathione disulfide is structurally close to both the metal(II)-bis-glutathionato conjugates (GS-M-SG) and to the lanthionine-bound glutathione thioether dimer (G-S-G) produced by nucleophilic attack of the thiolate group of glutathione to the electrophilic carbon of dehydro-alanine (acrylate) glutathione, as shown in Scheme 10.

In particular, this latter product has been identified in cells treated with busulfan and is a chemically stable structural analogue of glutathione disulfide, of which it does not have the reducible disulfide bond but retains the structural motifs for recognition by the glutathione disulfide-binding site of the enzyme. On the contrary, the metal(II)-bis-glutathionato conjugates retains the structural motifs for recognition by the glutathione disulfide binding site of the enzyme but are able to kinetically exchange the metal with other thiolates, through the mechanism reported in Section 2. This mechanism may explain the inhibition of the enzyme activity of several enzymes with essential catalytic or regulatory thiol groups by the thiol binding metals.

### 5.2. Glutathione Reductases

**Glutathione reductases (GR)** link the NAD(P)H pool to maintenance of the intracellular reduced glutathione pool by directly reducing GSSG to GSH by consuming NAD(P)H. The large, dimeric enzyme operates through a FAD cofactor and two essential cysteines, one of which is proximal to the glutathione disulfide docking site and the other to the FAD cofactor [129]. The details of the multistep mechanism postulated for the reaction and the structural motifs of the several enzymes, which have been thoroughly investigated, cannot be described in detail. Briefly, glutathione disulfide is held in the proximity of the catalytically active reducing thiol group and one thiol glutathione unit is detached in the first of the two reductive steps, leaving the enzyme in a cysteine-glutathionylated form. The intermediate form of the enzyme is further reduced by the formation of an intra-molecular disulfide bond between the two active site cysteines, leaving the enzyme in an “oxidized” form, from which the vicinal thiols are regenerated by the FAD(H)-intermediated hydride transfer from NAD(P)H.

**Scheme 11:**
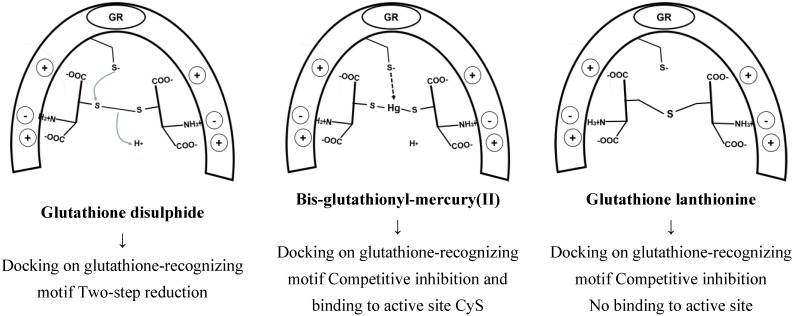
Competitive inhibition of the glutathione disulphide (GSSG) binding site by G-S-G and by the metal glutathione conjugate, exemplified by the active site of glutathione reductase.

In principle, this complex catalytic cycle can be disrupted at two levels by dehydro-alanine (acrylate) glutathione. A first mechanism is the direct interaction with the reducing thiolate, leading to irreversible inactivation of the active site, as described for the inactivation of GSTs (Scheme 9). A second mechanism is based on the competitive inhibition of the GSSG binding site by G-S-G and by the metal glutathione conjugate, as depicted in Scheme 11. The consequence of competitive or irreversible inhibition of GR is a decrease of reduced glutathione levels.

### 5.3. Glutaredoxins

**Scheme 12:**
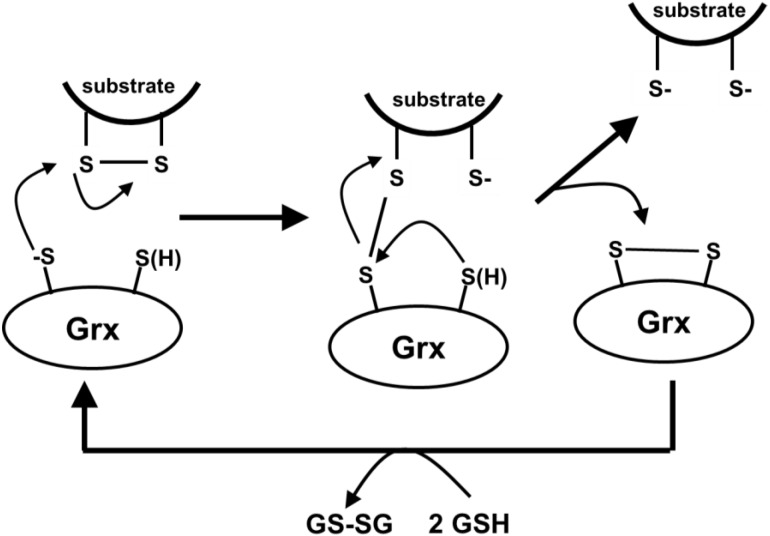
Mechanism of the trans-sulfuration reaction, as exemplified for the reduction of an intramolecular disulphide bond in a substrate protein, catalyzed by a two-thiol glutaredoxin.

**Glutaredoxins (Grx)** are the glutathione-dependent enzymes that catalyze the inter-conversion of soluble thiols and disulfides (trans-sulfuration) and the transfer of glutathione, cysteine, homocysteine and cysteamine groups to protein cysteines and from thiolated cysteines, as part of the biological control mechanisms. This process is kinetically slow under biological conditions of neutral pH, due to the very low concentration of the free thiolate groups, which are the true active agents of the reaction, as depicted in Scheme 12.

Since all Grx isoforms have a solvent-accessible free cysteine and a glutathione recognition motif, they may be targets for irreversible inactivation by dehydro-alanine (acrylate) glutathione.

To understand the toxicological activity of substances that are capable to generate dehydro-alanine (acrylate) glutathione, it is of a fundamental importance to consider the number of copies of the enzymes in cells. In yeast, it has been established that they are in the few thousand per cell (ScGrx3: 1.1 × 10^4^; ScGrx4: 7.8 × 10^3^; ScGrx5: 6.3 × 10^3^; ScGrx6: 1.6 × 10^3^ molecules per cell), so a very low number (tens of *zepto-*moles; one *zepto-*mole being *approx.* 600 molecules) [140]. Although similar information is not available for mammalian cells, these finding points at the possibility that even low levels of intracellular dehydro-alanine (acrylate) glutathione produced can inactivate a substantial fraction of the cellular Grx content. This is a very important and possibly crucial biochemical mechanism, since reversible thiolation of proteins can modify their enzymatic activities (glycolytic enzymes, components of the respiratory chain, creatine kinase, carbonic anhydrase), ion and metabolite transport, gene transcription, signal transduction and cell death.

### 5.4. Glutathione Peroxidases

**Glutathione peroxidases (Gpx)** are selenium-containing enzymes that degrade hydrogen peroxide and hydroperoxides to water and to alcohols, respectively, by using an active site seleno-cysteine that is transformed into the selenenic (Se-OH) oxidized form and is reduced back to the selenolate (Se-H) form by glutathione. Functionally analogous are the **Peroxiredoxins (Prx)**, which use the same mechanism but with a cysteine active site that shuttles from the thiolate (S-H) to the sulphinic acid (S-OH) form and is reduced back by glutathione. Both classes of enzymes are vulnerable to inactivation by electrophiles such as dehydro-alanine (acrylate) glutathione when they are in their reduced form.

### 5.5. Other Enzymes, Receptors and Regulatory Proteins

Reversible glutathiolation has been recognized as a regulatory mechanism for several enzymes, receptors and regulatory proteins. Some of these cellular targets are collected in Table 1. The relevance of this incomplete list is to highlight that irreversible inactivation, rather than reversible regulation, can occur through the reaction of the same redox-activated control motifs (cystine thiol(ate) residues) with EdAG, rather than with the physiologically appropriate actuator (glutathione sulphinate, glutathione disulphide).

**Table 1:** A list of enzymes, receptors and regulatory proteins for which the literature reports evidence or hint that they are reversibly glutathionylated and/or are substrates for glutaredoxins (Grx) (re-elaborated and integrated from Deponte [129], Copyright 2013, Elsevier B.V., and Kil and Park [141], Copyright 2005, American Society for Biochemistry and Molecular Biology).

Enzyme	Consequences of *S*-glutathionylation
glycolytic enzymes	alters enzymatic activity
glyceraldehyde-3-phosphate dehydrogenase	inactivated by S-glutathionylation
isocitrate dehydrogenases (ICDHs1; EC 1.1.1.41 and EC 1.1.1.42)	regulated by S-glutathionylation
creatine kinase	inactivation
carbonic anhydrase III	reversible regulation of phosphatase activity
components of the respiratory chain	formation of ROS
	reversible glutathionylation of complex I increases mitochondrial superoxide formation
Actin	polymerization and effect on cytoskeleton
membrane receptors, transporters and ion channels	alters ion and metabolite transport
	causes cell death
several protein kinases and phosphatases	alters signal transduction
protein kinase C (12), guanylate cyclase	alters enzymatic activity
nuclear factor I	alters signal transduction
Ras	alters signal transduction
c-Jun	redox-regulated by mechanisms that include protein *S*-thiolation
ubiquitin-activating enzymes	when cells are exposed to oxidants are glutathionylated, with a concomitant decrease in the ubiquitinination pathway
NFκB	redox-induced inhibition of DNA binding; cell death

In particular, the Krebs cycle is the central process of aerobic cells and produces both metabolic energy as ATP and reducing equivalents as NAD(P)H from the mineralization of organic carbon substrates channeled into the process in the form of acetyl-CoA. NADP-dependent isocitrate dehydrogenase is a key enzyme of the Krebs cycle which produces NADP by oxidative decarboxylation of isocitrate to α-keto-glutarate. This product is further processed to fumarate by oxidative decarboxylation. Fumarate itself is a Michael acceptor, and the covalent attachment to protein cysteines corresponds to the post-translational modification known as succination [142] and fumarate has been recently demonstrated to yield a glutathione thioether in *in vitro* treated cells [142]. NADP-dependent isocitrate dehydrogenase is inactivated by glutathionylation of a specific residue of cysteine by glutathione disulfide, which binds to a regulatory cysteine residue by trans-sulfuration.

Enzyme inactivation is not the only mechanism by which reactive electrophilic analogues of glutathione, such as EdAG, can elicit, disturb or disrupt biological mechanisms. Among the key regulatory protein which may be targeted by the dehydro-alanine glutathione analog are Keap1 Nrf2, the sensing cysteine thiolate of which can be chemically modified by electrophiles (such as the lipid-derived HNE) and triggers the transcription of genes for antioxidant proteins and for the intermediate metabolism [143].

Thus, the formation of the dehydro-alanine glutathione species (that observed at *m*/*z* 272 in the spectra of glutathione-bound metals) is a key to the possible explanation of the ability of metals such as lead and cadmium to generate oxidative stress in cells without the necessity that the electrochemically silent metal itself change its oxidation state.

A close structural analogue of EdAG is the lanthionine cross-link generated upon chemical stress in some structural proteins such as those of the eye crystalline, of bleached hair, of wheat glyadin. This post-translational motif is often generated from the β-elimination of phosphorylated serine residues in proteins and in some other instances from oxidized cysteines. Of a particular interest is the observation of an EdAG motif in the seleno-cysteine active site of RBC peroxyredoxin [144,145].

Further cysteine-rich protein motifs that can be modified by electrophiles such as EdAG are the Zn-binding Cys_2_His_2_ and Zn_2_/Cys_6_ domains (“*zinc finger*”) of regulatory proteins such as nuclease enzymes, of DNA transcription factors and of metallothioineins [146,147].

## 6. Metallothioneins and Their Conjugates with Arsenic, Cadmium, Mercury and Lead

Metallothioneins (MTs) are a family of small proteins which are widely distributed among the living organisms and contain 61–68 amino acids with an unusually high (>20) number of cysteine residues, present both in the disulfide and in the free thiol form. MTs react with and enhance the detoxification of a number of metals including zinc, mercury, copper and cadmium. MT-1 (according to the older classification) is the most functional and active MT in humans. Several MT-metal conjugates can be isolated from the tissue of animals exposed to several metals.

In the “native” state (*i.e.*, in the absence of excess exposure to metals), the free thiol groups of MTs usually bind the Zn^2+^ ions of the large and tightly regulated pool of the organism. In the presence of free ions of metals with a higher binding affinity, such as Cd^2+^ or Hg^2+^, or of metal ions in conjugate forms, such as the glutathione-Cu(I) [148] and the glutathione-Cd [149] conjugates, these metals bind MT thiol groups and the bound Zn(II) is released [150]. Released Zn^2+^ ions thus binds fairly similar domains (Zn-fingers) of other regulatory proteins (transcription factors), which in turn trigger signaling cascades leading to promote the synthesis of proteins useful in facing the effects of metal-mediated oxidative stress [151].

**Scheme 13:**
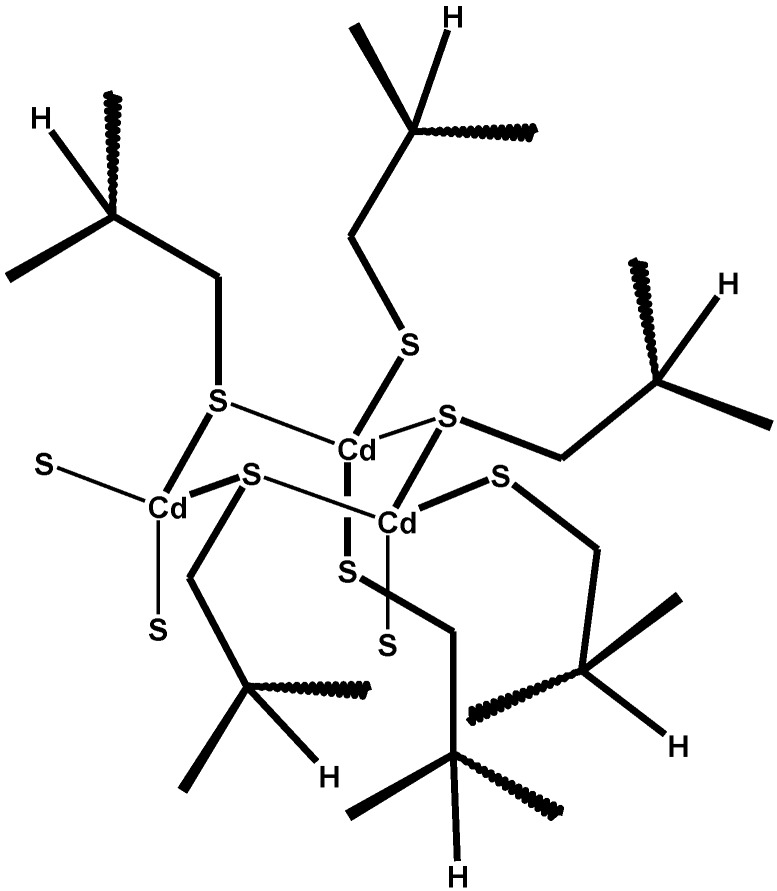
A schematics of the (Cd_3_S_9_) metal-thiolate motif of metallated metallothioneins (redrawn and adapted from Reference [152]. Copyright 1980, National Academy of Sciences; shown are six cysteine residues participating in the formation of the Cd(SCy)_2_ conjugate, while of those yielding coordinative bonds only the sulphur atoms are drawn).

According to the several studies conducted with different instrumental techniques, such as by Nuclear Magnetic Resonance (^113^Cd-NMR [152]), X-ray diffraction, and molecular mechanics and dynamics calculations [153], metallated metallothioneins look like protein chains wrapped around a metal cluster, with multiple cysteines liganding metals through their thiolate groups [154], as exemplified in Scheme 13 for the tetra-metallated domain of a Cd-loaded MT.

In this depiction, each Cd(II) ion is tetra-coordinated by four cysteine ligands: two are deprotonated to yield (formally) Cd-thiolate conjugates that are similar in principle to those of glutathione and of other soluble thiols, while other two complete the coordination sphere. The protonation state of the latter is not strictly necessary to impart to the metal-thiolate core its electrical charge (calculated as −3 [155]) and can be changing. This simplified picture, of course, does not take into account the possibility of a fast proton transfer between the 11 residues of cysteine. As will be highlighted in the following, this can play a role in the proposed explanation of the important biological role of metallated MTs in several organs where the accumulation of metals over lifetime ultimately generates its functional failure or the onset of cancer.

In particular, glutathione and MT conjugates of several metals are formed in the olfactory epithelium and are transported by pinocytosis by the olfactory axons to the brain, thus evading the blood-brain barrier (BBB) [156]. Among the metals for which this mechanism has been demonstrated are: zinc [157], cadmium [149,158,159], mercury [160,161], manganese [159,161,162,163], cobalt [164], nickel [165]. The speciation of metal at different times following *in vivo* exposure shows that cadmium, mercury initially form low-MW metal conjugates with glutathione, from which the metal is later transferred to MT. The metal-loaded proteins are then transported in a retrograde direction from the olfactory axon to the cell nucleus [149].

The overload of rats with zinc chloride yields a characteristic red fluorescence of the tissue, a phenomenon that intensifies when selenium (as sodium selenite) is co-administered. The metal-containing fraction of liver tissue, analyzed by X-ray diffraction, yielded reflection signals characteristic for cubic (zinc blende), zinc sulfide (ZnS), and/or zinc selenide (ZnSe) [166]. Co-administration of cadmium chloride and sodium selenite allows us to harvest the corresponding sulphide and selenide of cadmium both from the liver [167] and from the kidney [168]. The authors consider this phenomenon “*as a green biosynthesis of nanoparticles*”, and biological techniques that exploit plant cells and micro-organisms and for the biological production of metal sulphide nanoparticles are currently evaluated as an economically competitive and environmentally benign alternative to “chemical” processes [169,170,171,172,173].

That Cd-Mt is the main form of the metal accumulated in the liver and in the kidney of experimentally intoxicated rats has been recently demonstrated by XANES and EXAFS [174]. In human kidneys transplanted but rejected from the receiving subject, almost all of the metal in tissue was in the form of cadmium-metallothionein [175]. Also, the body burden of cadmium, mercury and lead in the cortex is not associated to the level of known soluble biomarkers of oxidative stress, such as 8-oxo-deoxyguanosine [176]. However in the tissue of human kidney tumors, the level of cadmium is much higher in the tissue surrounding the tumor than in the tumor tissue itself, but the immuno-reactivity of (apo)-metallothionein in the latter is negligible, thus pointing at a different carcinogenic form of cadmium than that embedded in MT [177].

In addition, histochemical studies performed by auto-metallography [178,179,180] show that in some organs metals can accumulate as sub-micrometre sized particles of elemental gold [181] and silver [178,182], of the sulphides of mercury [183,184,185,186,187,188] and of bismuth [180,189].

Very small crystals (*i.e.*, sized 2–10 nanometre, or *approx.* the diameter of 50 atoms) of some metal chalcogenides, such as cadmium sulphide, cadmium selenide, arsenic telluride, show specific optoelectronic properties. In particular, these semi-conductor materials can absorb UV radiation and re-emit light in the visible range with wavelengths that increase as the dimension of the crystal increases: smaller dots appear blue; the color of larger ones is progressively red-shifted. These materials find several applications in contemporary consumer technology, such as in the color screens of portable IT devices, and their now widespread use has raised toxicological concern [190,191]. For specially engineered nanoparticles there are also potential uses for medical diagnosis and therapy at the cellular level, such as to target, highlight and possibly kill cancer cells [192]. It is of some interest that realgar, a polymorph of arsenic sulphide (As_4_S_4_), and cinnabar (mercury sulphide) found an ethno-pharmacological use in Chinese medicine [193,194].

Among the mechanisms envisaged to occur to explain their toxicity against cells in culture are the release of toxic cadmium ions through their dissolution by the acidic environment of cell lysosomes [195] and their ability to generate and trap free radicals [196,197,198,199].

The generation of nanoparticles of cadmium sulphide and of other metal chalcogenides by living cells such as bacteria [200] and plants does not only occur from metallothionein but also from other analogue metal-protein conjugates, such as phytochelatins.

It is thus of relevance to understand whether the metal core of metallothioneins can originate metal sulphide nano-crystals from the β-elimination mechanism and whether these nano-crystals can play a role in the organ toxicity of accumulated metals, especially in the kidney. Some information derives from a limited number of studies performed on different types of already commercially available semiconductor quantum dots constituted of homogeneous cadmium sulphide (CdS), of Cadmium selenide (CdSe) and of a cadmium selenide core coated with an outer shell of zinc sulphide (CdSe/ZnS). In one research [197], the surface of the CdS nanoparticles is covered (“*capped*”) with mercaptoacetic acid (the thiolate group of which binds the cadmium ions of the outer layer) and they are irradiated with UV light in the presence of DMPO, a free radical spin trap probe. EPR spectroscopy allowed recognizing the generation of reaction products of DMPO that derive from its reaction with superoxide (in its turn, recognized from its disappearance from the reaction mixture after incubation with superoxide dismutase). On the contrary, CdSe and CdSe/ZnS nanparticles did not generate free radicals.

In another study [198] CdSe nanoparticles capped with a lipophilic phosphine were incubated with a dimeric radical probe consisting in a mercaptoacetic disulphide unit linked to two TEMPO-derived units. The addition of the nanoparticles caused the homolitic fission of the mercaptoacetic disulphide bond, even in the absence of irradiation. In addition, the process of fission of the disulphide bond quenched the natural fluorescence of the nanoparticle.

The production of free radicals at the surface of the nanoparticles depends both on the composition of the core and on the nature of the capping groups on its surface. One study [199] was aimed at understanding whether metallothioneins can capture metal ions released from CdSe nanoparticles. One peptide with 10 cysteine residues, the sequence of which mimics that of the α-domain of MT, binds the nanoparticles and a mass spectrometric analysis of the peptide-bound nanoparticles shows that it binds three cadmium ions released from the surface.

**Scheme 14:**
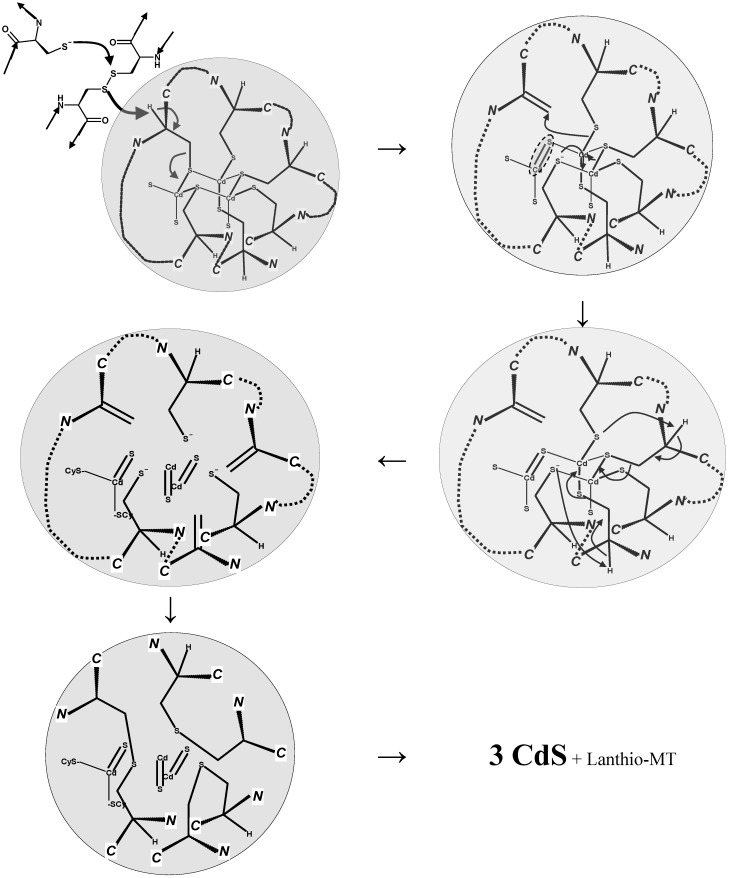
Generation of cadmium sulphide units from the Cd_3_S_9_ unit (Cluster B) of Cd-metallothionein.

The generation of cadmium sulphide and of lanthionine-linked units from metal-laden metallothionein can occur by a sequence of β-elimination steps in a domino series as exemplified for the core Cd_3_S_9_ unit of Cluster B in Scheme 14.

In the exemplified reaction mechanism, the triggering nucleophile is a thiolate group produced by trans-sulphuration of a disulphide bis-cysteine group of MT. The several cysteines in MTs are in part in the metal-binding thiolate form and in part as intramolecular disulphide groups [201,202]. The latter form can act as a responder element sensitive to the intracellular redox status which also helps to determine the intracellular localization of the protein [203,204,205]. The intramolecular thiolate group is released from its disulphide-bound form by reaction with a thiolate group from cysteine or glutathione and the intramolecular nucleophile triggers the reaction cascade leading to the formation of three lanthionine-bound molecular units within the amino acid sequence of MT and of three units of cadmium sulphide. The latter can coalesce into a wurtzite elemental cell and add up with others to generate a nano-crystal of cadmium sulphide.

The presented mechanism, that uses a thiolate group as intramolecular base, is among those possible, one that has an analogy (however remote) with one that has already been recognized as biochemically occurring. In the coagulation cascade, a key step is the conversion of prothrombin into the active form by carboxylation of glutamic acid to γ-carboxy-glutamate. This reaction entails the abstraction of the C-4 proton in the side chain of glutamic acid and reaction of the resulting carbanion with activated carbon dioxide. The strong protic base necessary to abstract the proton is a tertiary alkoxide that is generated from the hydroquinone form of vitamin K, through a mechanism involving the reaction with molecular oxygen, referred to as “*base strength amplification by oxidation*” [206].

## 7. Conclusions and Perspectives

The mechanism through which some metals such as cadmium are able to produce human cancer and to generate oxidative stress, even if they cannot generate noxious free radicals through the general Fenton pathway, has so far escaped a chemically based explanation. That illustrated in this article is a further approach to achieve such results, and is obtained through a merge of the information derived from several different fields of investigation into the properties of metal-thiolate systems of biological interest.

First, binding of ions such as Mercury(II) and Arsenic(V) to thiols allows two-electron oxidation of the thiol through the reduction of the metal center without the generation of radicals. In the case of Mercury, this mechanism can be catalytic, due to the fast and efficient intracellular oxidation of Mercury atoms, and lead to the depletion of the soluble thiolome. Moreover, the same mechanism can lead to the inactivation of enzymes that use vicinal thiol groups as active sites or as regulatory elements. Both these mechanisms can explain the much higher toxicity of mercury, when compared to that of other thiol-binding metals that cannot sustain an autocatalytic reversible redox cycle.

A more general mechanism to explain oxidative stress generated by electrochemically silent metals is based on the formation of glutathione conjugates and on their decomposition under physiological conditions to yield two toxic agents: the nano-particulate metal sulphides that deposit in organ parenchyma and the electrophilic dehydroalanine analogue of glutathione. Each of these reaction products is able to produce own harmful effects on several biological targets. In particular, the peculiar endogenous electrophilic motif of dehydrolalanine can be generated not only from organic glutathione thioethers and from thiol-metal conjugates, but also in proteins from phosphorylated serine residues. The occurrence of lanthionine motifs generated in bio-structures by intramolecular covalent attachment of cysteine or by reaction with glutathione has been identified as a marker of protein aging [62,126,207,208,209]. The general occurrence of this dehydroalanine-derived post-translational modification thus makes a strong candidate to search by proteomic-based approaches [210] its presence in inactivated enzymes and in other proteins. To be able to relate the presence of this signature to the exposure to electrochemically silent toxic metals can strengthen the hypothesis of its role in a general mechanism based on induction of a condition of chronic oxidative stress- the somatic and carcinogenic effects of toxic metals and of other disease-generating conditions.

The formation of metal sulphides as detoxification forms of some thiol-binding metals has been long recognized in several organisms, from microorganisms and plants to mammals. Nano-particulate metal sulphides and chalcogenides feature peculiar opto-electronic properties that make these materials of current technological appeal and their production from organisms such as bacteria and plant cells in culture is currently being explored as a more environmentally friendly alternative to chemical production. In mammals, their generation from metal-loaded metallothioneins can occur by means of β-elimination processes involving the several binding cysteines, according to a chemical mechanism analogous to that leading to the decomposition of glutathione-metal conjugates and leading to the production of the dehydroalanine analogue of glutathione. It is thus likely that this is a major form of metal accumulation throughout the human life and that the ability of nano-sized crystals to generate reactive chemical species under irradiation with light may possibly be related to their activity as catalytic generators of free radicals when embedded into the living tissue.

Although several steps in this proposed general mechanism still need detailed confirmation in *ex vivo in vitro* and in *in vivo* models through combined metallomic-metabolomic approaches [211,212] and through a systematic physico-chemical study of the involved molecular systems, the collected information allows us to design experimental approaches aimed at verifying the missing steps of the comprehensive model.

## Abbreviations

ATP: adenosine-triphosphate
BBB: blood-brain barrier
CAD: collision-activated decomposition (technique of mass spectrometry)
CAD-MS-MS: collision-activated decomposition tandem mass spectrometry (technique of mass spectrometry)
DMPO: 5,5-Dimethyl-1-pyrroline *N*-oxide (a spin-trap compound)
EdAG: γ-glutamyl-dehydroalanyl-glycine
ERMS: *Energy-Resolved (tandem) Mass Spectrometry* (technique of mass spectrometry)
EXAFS: extended X-ray absorption fine structure (spectroscopic technique)
FAD: flavin adenine dinucleotide, oxidized form
GCL: glutamate-cysteine-ligase (holo-enzyme)
GGT: γ-glutamyl-transpeptidase (enzyme)
GIBMS: Guided-Ion Beam Mass Spectrometry (technique of mass spectrometry)
GLCL: glutamate-cysteine-ligase (functional enzyme sub-unit)
GLCR: glutamate-cysteine-ligase (regulatory enzyme sub-unit)
Gpx: Glutathione peroxidase (enzyme/s)
GR: Glutathione reductase (enzyme/s)
Grx: Glutaredoxins (enzyme/s)
GSH: glutathione (γ-ECG), reduced form
GSSG: glutathione disulphide
GST: glutathione*-S-*transferase
GSTA1-1: glutathione*-S-*transferase isoenzyme A-A1
IARC: International Agency for Cancer Research
MerA: mercuric reductase A (enzyme)
MMA(V): mono-methyl-arsonic acid (V)
MS-MS: tandem mass spectrometry
MTs: Metallothioneins
NAC: *N-*acetyl-cysteine
NADH: nicotinamide adenine dinucleotide, reduced form
NADPH: phosphorylated nicotinamide adenine dinucleotide, reduced form
NMR: Nuclear Magnetic Resonance (spectroscopic technique)
Prx: Peroxiredoxins (enzyme/s)
RBC: red blood cells (erythrocytes)
ROS: Reactive Oxygen Species
TBARS: thiobarbituric acid reactive substances (a class of organic electrophiles)
TEMPO: 2,2,6,6-Tetramethylpiperidine-1-Oxyl (a spin-trap compound)
XANES: X-ray absorption near edge structure (spectroscopic technique)

## Dedication

I dedicate this article to the beloved memory of my parents, Letizia (*née* Morabito, 1929–2009) and Giovanni Maria Rubino (1925–2013) for all their encouraging love and guidance, and of my own early mentor, Professor Bruno Danieli (1939–2014) of the Università degli Studi di Milano, for much personal friendship and intellectual support.
